# Influence of Aging and Gender Differences on Feeding Behavior and Ghrelin-Related Factors during Social Isolation in Mice

**DOI:** 10.1371/journal.pone.0140094

**Published:** 2015-10-08

**Authors:** Chihiro Yamada, Yayoi Saegusa, Miwa Nahata, Chiharu Sadakane, Tomohisa Hattori, Hiroshi Takeda

**Affiliations:** 1 Tsumura Research Laboratories, Tsumura & Co., Ibaraki, Japan; 2 Pathophysiology and Therapeutics, Faculty of Pharmaceutical Sciences, Hokkaido University, Sapporo, Hokkaido, Japan; 3 Hokkaido University Hospital Gastroenterological Medicine, Sapporo, Hokkaido, Japan; University of Santiago de Compostela School of Medicine - CIMUS, SPAIN

## Abstract

Psychological stress due to social isolation is known to cause abnormal feeding behaviors, but the influences of gender and aging on subchronic stress-induced changes in feeding behaviors are unknown. Thus, we examined the changes in body weight, food intake, and orexigenic ghrelin-related factors during 2 weeks of isolation stress in young and aged mice. Food intake increased significantly in young mice in the isolation group compared with the group-housed control throughout the experimental period. This isolation-induced increase in food intake was not observed in aged mice. In young mice, there were no significant differences in body weight between the isolated group and group-housed control up to 2 weeks. However, aged male mice exhibited significant weight loss at 2 weeks and a similar tendency was observed in aged female mice. Young male mice, but not female mice, had significantly increased (2.2-fold) plasma acylated ghrelin levels after 1 week of isolation compared with the group-housed control. A significant but lower increase (1.3-fold) was also observed in aged male mice. Hypothalamic preproghrelin gene expression decreased significantly with isolation in young male mice, whereas it increased significantly in female mice. The expression levels of NPY and AGRP in the hypothalamus, which are transmitted by elevated peripheral ghrelin signals, increased significantly in isolated young male mice, whereas the AGRP expression levels decreased significantly in young female mice. Isolation caused no significant differences in the expression levels of these genes in aged mice. In isolation, young female mice exhibited markedly increased dark- and light-phase locomotor activities compared with male mice, whereas male and female aged mice exhibited no obvious increases in activity immediately after the dark phase started. We conclude that the gender-specific homeostatic regulatory mechanisms required to maintain body weight operated during subchronic psychological stress in young mice but not in aged mice.

## Introduction

In addition to psychological reactions such as depression and anxiety, stress triggers various physiological reactions including an increase in respiration and blood pressure. In particular, changes in feeding behavior are well-known phenotypes related to stress.

Acute stress loading and direct activation of the corticotropin-releasing factor (CRF) receptor in experimental animal models clearly decrease food intake [1–4], but chronic psychological stress does not necessarily have a negative impact on feeding behavior. For example, obvious hyperphagia [5], weight gain [6, 7], or a preference for palatable food have been observed in a chronic social defeat model in rodents. These phenomena may be a type of stress adaptation reaction to chronic stress [8].

Ghrelin, a hormone produced in the peripheral organs and central nervous system, is known to be involved in the regulation of appetite, gastrointestinal motility, and growth hormone release. Recently, the abnormal kinetics of ghrelin due to stress loading have been reported [1–3, 9, 10]. Intracerebroventricular administration of CRF or exposure to psychological stress suppresses food intake in association with decreases in peripheral ghrelin levels during fasting [1, 3]. In ghrelin deficienct conditions, the administration of exogenous acylated ghrelin or the endogenous ghrelin enhancer rikkunshito clearly recovers the effect of stress-induced hypophagia [1–4, 6, 10]. In contrast to the negative impact of acute stress on feeding and ghrelin kinetics, the food intake and peripheral ghrelin levels were shown to increase in chronic stress models such as water immersion [11] and social defeat [5, 12, 13]. These findings indicate that increased ghrelin signaling plays a critical role in the overeating caused by chronic stress.

Loneliness and the lack of social networks in the elderly are important risk factors for the onset or recurrence of depression [14]. Aging changes the secretion and reactivity of neurotransmitters that affect stress response and adaptation [3, 4, 15, 16], as well as the expression of glucocorticoid receptors [17]. Aging also appears to have a major influence on the sensitivity to acute psychological stress responses [3, 4, 17]. There are also gender differences in the sensitivity to acute stress responses, where clinical depression tends to be more common among women; and experimental stress is related to model type-dependent gender differences [18, 19]. However, the effects of aging and gender on feeding behavior during subchronic isolation stress have not been studied previously.

In the present study, we clarified the age- and gender-specific effects of subchronic isolation on body weight, food intake, the plasma ghrelin level, locomotor activity, and the expression of feeding- and stress-related genes in the hypothalamus of mice. Based on the results obtained in this study, we propose hypothetical mechanisms that underlie gender- and age-related differences in the regulation of feeding behaviors in isolated conditions.

## Materials and Methods

### Animals

**Fig 1:**
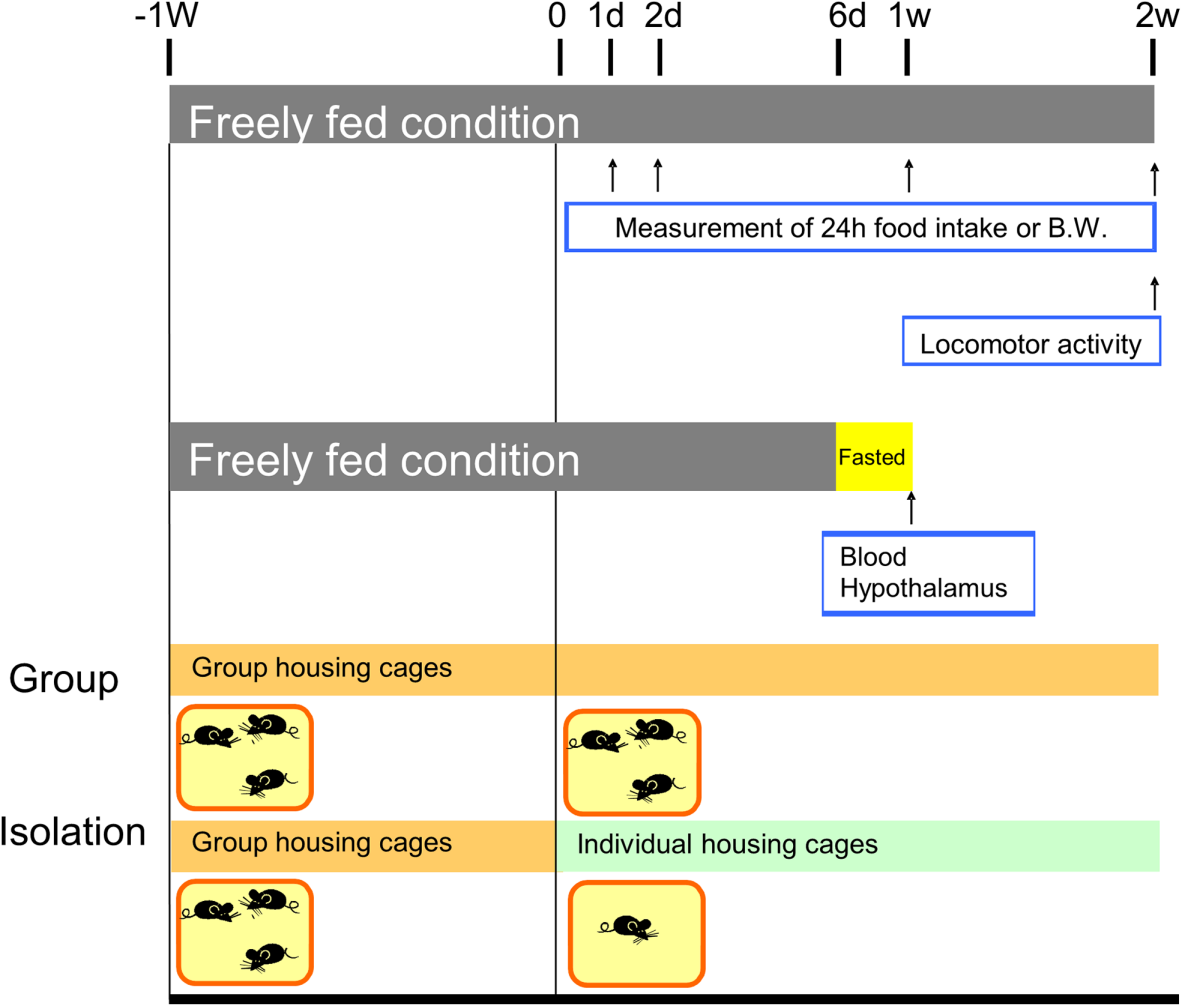
Experimental protocol employed in this study.

Six- to 10-week-old and 79- to 91-week-old C57BL/6 mice (Charles River. Co. Ltd., Japan) were used as young and aged mice, respectively. Before the experiments, mice were acclimated in cages (3–4 mice/cage) in a temperature- and humidity-controlled room with a 12-h light/dark cycle, and with free access to food and water for more than at least 1 week. After acclimation with 3–4 animals per cage, the young and aged mice were each divided into two groups. The mice that received the group-housed control treatment were kept with 3–4 animals per cage; whereas, the mice in the isolated housing group were caged individually starting from the dark period (Fig 1). The mouse isolation was performed by transferring group-housed mice (3–4 mice/cage) to separate cages (1 mouse/cage) and evaluated the each parameter during 2 weeks. This study was approved by and conducted according to the guidelines of the experimental animal ethics committees of Tsumura & Co. (Ibaraki, Japan; permit no.: 08–212, 09–178, 12–105, 13–002).

### Body weight and food intake

The body weight of each mouse was measured on the day of isolation, and after 1 day, 1 week, and 2 weeks of isolation. The body weight increase rate was calculated as the increases during 0–1 days (1 day), 0–1 week (1 week), and 0–2 weeks (2 weeks) after starting isolation. The food intake was measured after 2 days, 1 week, and 2 weeks of isolation. For the group-housed mice, the total food intake per cage was divided by the number of animals to calculate the food intake per animal for each cage [1, 4], because food consumption of individual mice within group housing (3–4 animals per cage) is impossible to measure. Therefore, there is a case where the minimum of the animal number is 3 (That is, total mice data for at least 9 aged mice were included) with respect to body weight and food intake of aged animals. Further, the weight that is linked to food intake was the same as the average value for each cage.

### Locomotor activity

The locomotor activity of mice in the home cage was measured according to a previously described method [20, 21]. The locomotor activity was assessed for individual cages during experimental period using the infrared sensor NS-AS01 (Neuroscience Co. Ltd., Tokyo, Japan) and a DAS data recording and analysis system with free access to food. Locomotor activity from initiation to completion was regarded as one count. In the group-housed mice, the locomotor activity per animal was expressed by dividing the total counts per cage by the number of animals. For the isolated group, the locomotor activity was determined for each isolated animal. The data were quantified by dividing them into dark and light periods, which are the active and resting periods for mice, respectively, and by evaluating the social and isolated groups.

### Plasma hormone level

To determine the mechanism that underlies the effect of isolation rearing on food consumption, the mice were subjected to 18-h fasting for 1 week after starting isolation and blood was collected from the abdominal vena cava between 13:00 and 16:00 under isoflurane anesthesia. Animals were exsanguinated death after blood collection. EDTA-2Na and aprotinin were used during blood collection, and the collected blood was centrifuged immediately to collect the blood plasma. To determine the blood ghrelin level, 10% HCl (1 N) was added to the blood plasma and the levels of acylated ghrelin and des-acyl ghrelin were measured in the plasma (Active Ghrelin ELISA Kit/ Desacyl-Ghrelin ELISA Kit; LSI Medience Corporation, Tokyo). The ratio of acylated ghrelin relative to des-acyl ghrelin (A/D) was also calculated based on the value quantified for each component.

### Total RNA extraction and reverse transcription polymerase chain reaction (RT-PCR) analysis

The hypothalamus and stomach fundus strips were removed rapidly from each 18-h fasted mouse at 1 week after exposure to isolation after death from exsanguination under isoflurane anesthesia, and they were frozen immediately in a tube on dry ice. We used samples of the strips from the same site of gastric fundus. Isolated tissue homogenization and total RNA extraction were performed using an RNeasy Universal Tissue Kit (Qiagen, Valencia, CA, USA). Each sample was then diluted to 100 ng/μL. The diluted total RNA was incubated at 70°C for 5 min and then cooled on ice. Total RNA (1,000 ng) was reverse transcribed using a TaqMan Reverse Transcription Reagents Kit (Applied Biosystems, Foster City, CA, USA). Quantitative PCR assays were performed using TaqMan Universal PCR Master Mix (Applied Biosystems) with a Prism 7900HT Sequence Detection System (Applied Biosystems). The mRNA expression levels were normalized using ribosomal protein S29 as an endogenous control to correct for differences in the amount of total RNA added to each reaction. The differences were expressed as the dCt (Ct, threshold cycle) value, i.e., dCt = 2^(−|A−B|)^, where A and B are the number of cycles needed to reach the threshold by the housekeeping and target genes, respectively. All of the oligonucleotide primers and fluorogenic probe sets used for TaqMan real-time PCR were manufactured by Applied Biosystems (*Rps29*, Mm02342448_gH; *Htr1b*, Mm00439377_s1; *Htr2c*, Mm00434127_m1; *Crf*, Mm01293920_s1; *Mc4r*, Mm00457483_s1; *Ucn1*, Mm00445261_m1; *Ghrl*, Mm00445450_m1; *Ghsr*, Mm00616415_m1; *Npy*, Mm00445771_m1; *Agrp*, Mm00475829_g1; *Pomc*, Mm00435874_m1; *orexin*, Mm01964030_s1).

### Statistical analyses

A two-way repeated measures factorial analysis of variance (ANOVA) test followed by the Tukey–Kramer post hoc test were used to compare the results obtained for group-housed and isolated mice. The data obtained from the analyses of plasma ghrelin and hypothalamic gene expression levels and locomotor activity counts were compared using the Student’s *t*-test or Aspin–Welch’s *t*-test. The data were expressed as the mean ± standard error for each group, and *p* < 0.05 was considered statistically significant.

## Results

### Body weight and food intake

**Fig 2:**
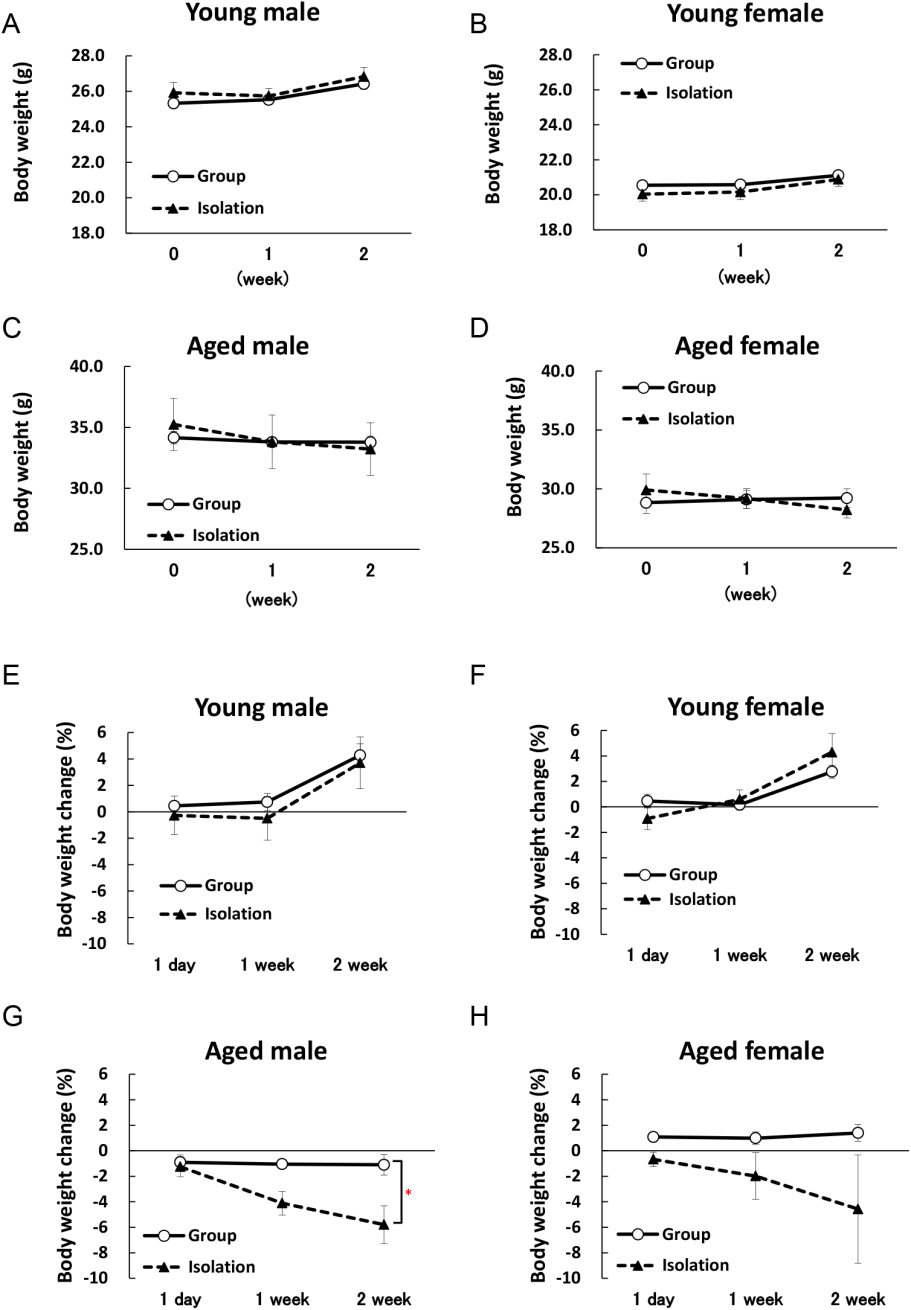
Body weight and changes in body weight during 2 weeks of isolation. Two-way repeated measures factorial ANOVA of body weight in all groups detected no significant changes. In the young male and female mice, two-way repeated measures factorial ANOVA of body weight changes detected no effects of isolation in males [young male: F(1, 33) = 0.4245, n.s.; young female: F(1, 36) = 0.0507, n.s.], significant effects of time in males and females [young male: F(2, 33) = 4.3506, p < 0.05; young female: F(2, 36) = 9.4196, p < 0.001], but the isolation × time interaction effects [young male: F(2, 33) = 0.0247, n.s.; young female: F(2, 36) = 0.9639, n.s.] were not significant. In aged mice, there were significant effects of isolation in males [aged male: F(1, 21) = 7.9029, p < 0.05; aged female: F(1, 24) = 2.0955, n.s.], time in males [aged male: F(2, 21) = 3.9764, p < 0.05; aged female: F(2, 24) = 0.4656, n.s.], but the isolation × time interaction effects [aged male: F(2, 21) = 1.7366, n.s.; aged female: F(2, 24) = 0.2593, n.s.] were not significant. Body weights of young male mice (A), young female mice (B), aged male mice (C), and aged female mice (D) are shown. Changes in the body weights of young male mice (E), young female mice (F), aged male mice (G), and aged female mice (H) are shown. The data represent the mean ± SEM (Young group: n = 5, Young isolation: n = 8–9, Aged group: n = 3, Aged isolation: n = 6–7).*p < 0.05 vs. group-housed mice.

In young mice, there were no significant differences in body weight (Fig 2A and 2B) or increases in body weight (Fig 2E and 2F) between the isolated group and group-housed control during 2 weeks of isolation. The body weights of stress-loaded aged male and female mice tended to decrease with time, although these differences were not statistically significant (Fig 2C and 2D). Aged male mice in the isolated group exhibited significant body weight loss at 2 weeks and a similar tendency was also observed in aged female mice (Fig 2G and 2H).

**Fig 3:**
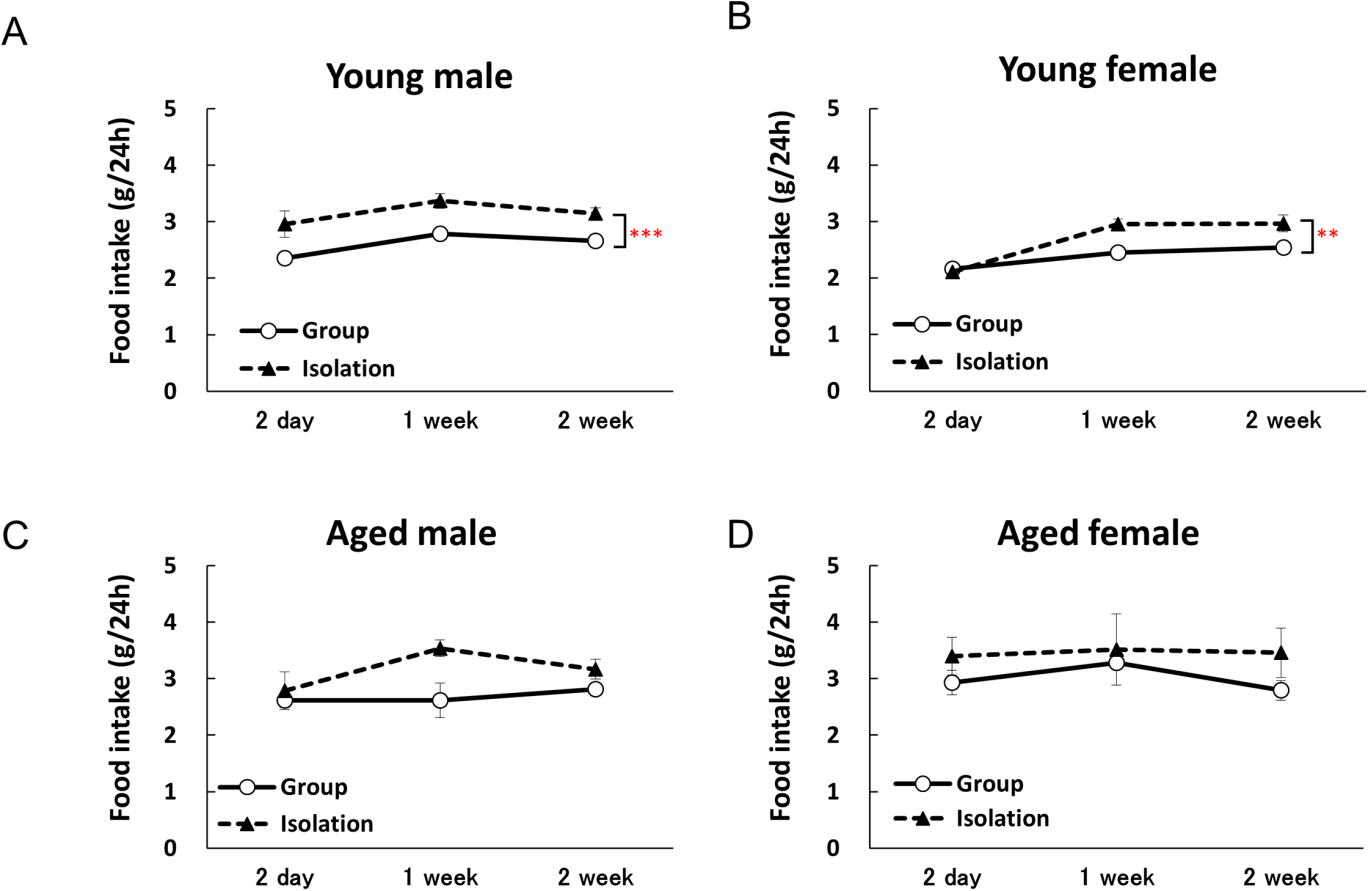
Food intake rates during 2 weeks of isolation. Two-way repeated measures factorial ANOVA of 24-h cumulative food intake in the young males and females detected significant effects of isolation [young male: F(1, 33) = 18.563, *p* < 0.001; young female: F(1, 36) = 7.9896, *p* < 0.01] and of time [young male: F(2, 33) = 3.6586, *p* < 0.05; young female: F(2, 36) = 20.315, *p* < 0.001], but the isolation × time interaction effects [young male: F(2, 33) = 0.0877, n.s.; young female: F(2, 36) = 2.7901, n.s.] were not significant. In aged mice, there were no significant effects of isolation [aged male: F(1, 22) = 4.1125, n.s.; aged female: F(1, 24) = 1.0384, n.s.] or of time [aged male: F(2, 22) = 1.8869, n.s.; aged female: F(2, 24) = 0.0923, n.s.], and isolation × time interaction effects [aged male: F(2, 22) = 1.1071, n.s.; aged female: F(2, 24) = 0.0793, n.s.] were not significant. The 24-h cumulative food intake rates in young male mice (A), young female mice (B), aged male mice (C), and aged female mice (D) are shown. The data represent the mean ± SEM (Young group: n = 5, Young isolation: n = 8–9, Aged group: n = 3, Aged isolation: n = 6–7). **, ***p < 0.01, 0.001 respectively vs. group-housed mice.

Young male mice in the isolated group exhibited significant increases in their 24-h food intake throughout the experimental period compared with the group-housed mice. In young female mice, there was a significant increase in food intake in the first week of isolation (Fig 3A and 3B). In aged mice, no significant difference in food intake was observed between the group-housed mice and isolated mice throughout the experimental period (Fig 3C and 3D).

### Plasma hormone levels and expression of stress genes in the hypothalamus

**Fig 4:**
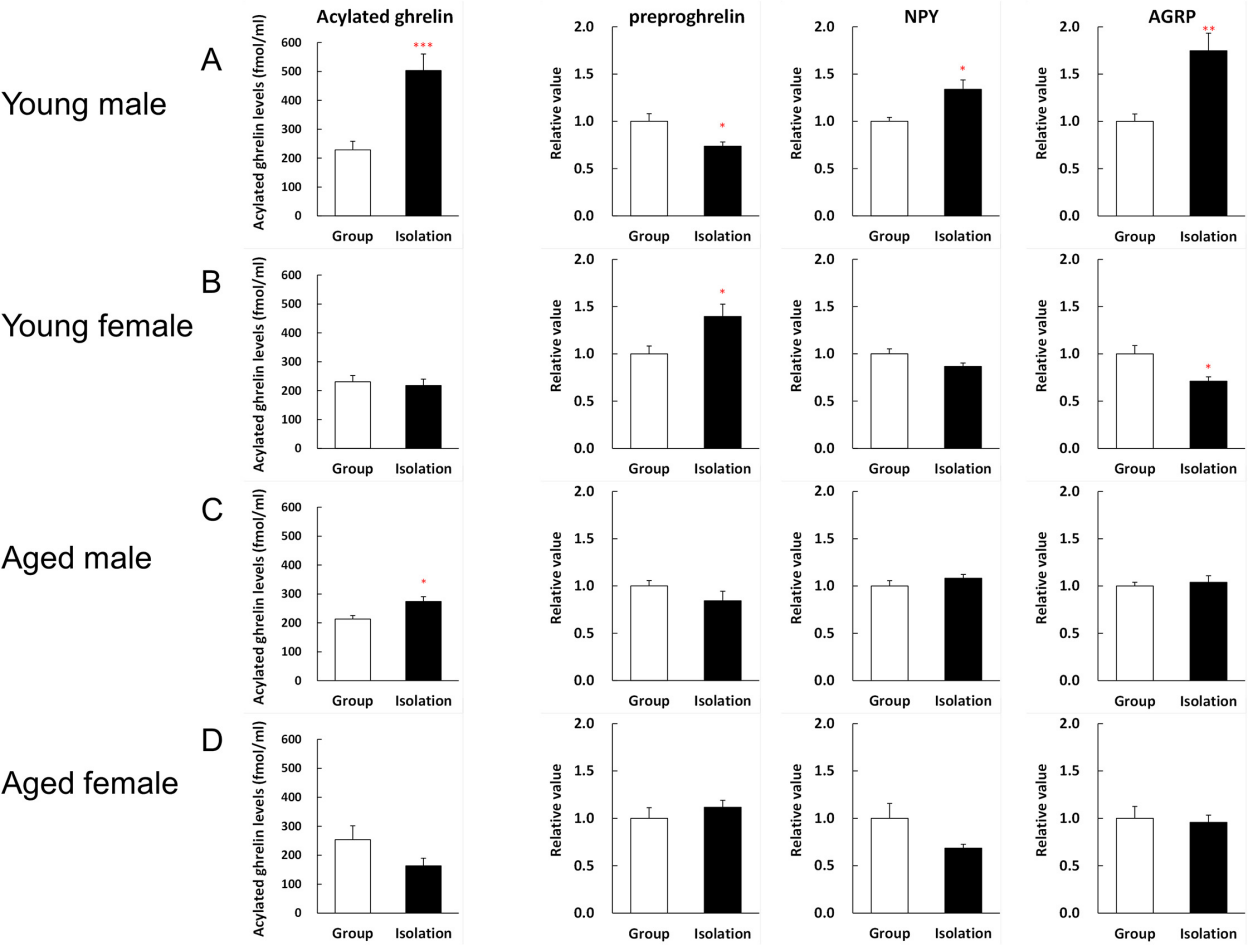
Changes in the plasma acylated ghrelin levels and hypothalamic gene expression levels. After 1 week of isolation, the mice were fasted for 18 h. The plasma acylated ghrelin levels and hypothalamic gene expression levels in young male mice (A), young female mice (B), aged male mice (C), and aged female mice (D) are shown. The data represent the mean ± SEM (Young group: n = 7–8, Young isolation: n = 8, Aged group: n = 4–5, Aged isolation: n = 7–8). *, **, ***, *p* < 0.05, 0.01, 0.001 respectively vs. group-housed mice.

As shown in Fig 4A, young male mice had significantly increased (2.2-fold) plasma acylated ghrelin levels after 1 week of isolation compared with the group-housed mice. The A/D ratio was also significantly higher in young male mice in the isolation group compared with the group-housed mice (1.7-fold) (S1 Fig). Similarly, there was a significant but lesser (1.3-fold) increase in isolated aged male mice (Fig 4C), but the A/D ratio did not differ between isolated and group-housed aged male mice (S1 Fig). The isolation of young or aged female mice had no effect on their plasma acylated ghrelin levels, but there were significant decreases in the A/D ratio in aged female mice when they were isolated. There were no significant differences in the des-acyl ghrelin levels of young and aged mice (data not shown).

As shown in Fig 4A, the expression level of the preproghrelin gene decreased significantly in the hypothalamus of isolated young male mice. The expression levels of the NPY, AGRP (Fig 4A), and orexin genes (S2 Fig) were elevated significantly. By contrast, the expression level of the preproghrelin gene was increased significantly by isolation in young female mice (Fig 4B). The AGRP gene expression level decreased significantly and the expression levels of the NPY (Fig 4B) and orexin genes (S2 Fig) tended to decrease in the isolated group. Isolation had no effect on the expression of the GHSR gene (S2 Fig). Isolation caused no significant differences in the expression levels of these genes in aged male and female mice, and aged female mice tended to have similar expression levels to those found in young female mice. Gender or aging had no effect on the expression levels of the preproghrelin, MBOAT, FOXO1, and PCSK1 genes in the stomach after isolation (data not shown).

The CRF gene expression level increased significantly in young male mice, but it decreased in young female mice and did not change in aged mice. Furthermore, in aged male mice, the expression level of the UCN1 gene decreased significantly in the isolated group, whereas there were no significant changes in the expression levels of other genes (S2 Fig).

### Locomotor activity during the second week of isolation

**Fig 5:**
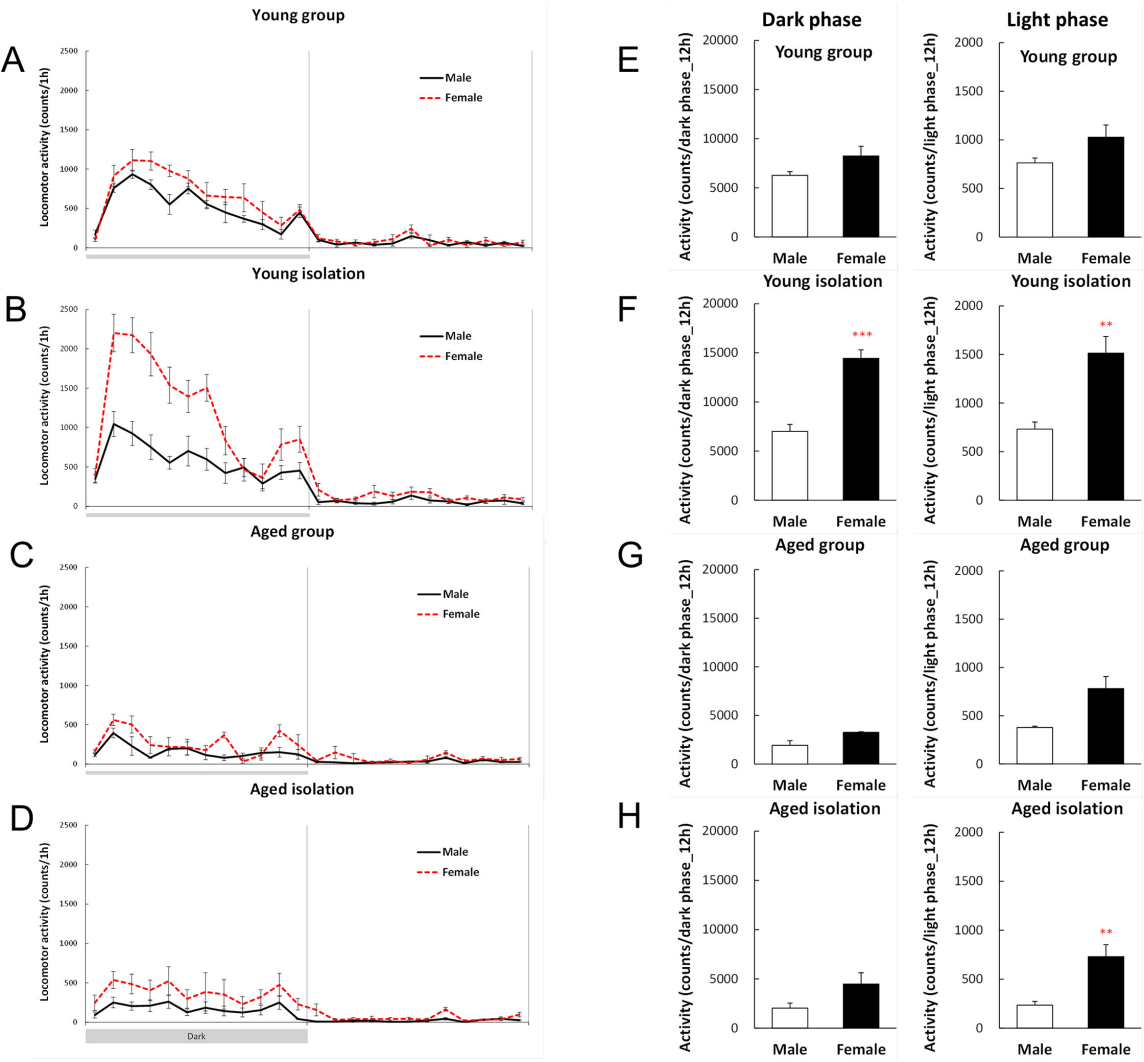
Locomotor activity levels after 2 weeks of isolation. The 24-h locomotor activity levels are shown for group-housed young mice (A), isolated young mice (B), group-housing aged mice (C), and isolated aged mice (D), as well as quantitative data for the locomotor activity levels (E–H). The data represent the mean ± SEM (Young group: n = 5, Young isolation: n = 8–9, Aged group: n = 3, Aged isolation: n = 6–7). **, ***p < 0.01, 0.001 respectively vs. male mice.

Body weight is regulated by the balance between food intake and energy expenditure, so we examined the locomotor activity during the second week of isolation. In both the male and female young mice, the locomotor activity in the group-housed mice increased rapidly immediately after the start of a dark phase, before decreasing gradually toward the light phase (Fig 5A). No gender differences were observed in terms of the dark and light phase locomotor activities in the group-housed mice (Fig 5A). However, the isolated young female mice exhibited marked increases in their dark phase and light phase locomotor activities compared with male mice (Fig 5F). In contrast to the activity patterns of young mice, male and female aged mice did not exhibit major increases in their activity levels immediately after the start of a dark phase (Fig 5C and 5D). Isolated aged female mice exhibited a slight but significant increase in their light phase locomotor activity compared with isolated aged male mice.

## Discussion

In the present study, we determined the effects of gender and aging on food intake and ghrelin-related parameters during 2 weeks of rearing in isolation.

We have previously reported that the novel environmental change caused by isolation suppressed food intake during the first several hours of isolation [1, 3]. In the present study, isolation rearing was extended for 2 weeks, thereby exposing mice to subchronic isolation. In these conditions, a significant increase in food intake was observed in young mice but not in aged mice. In the breeding environment of group-housing, it is well known that hierarchical relationships between mice can affect nutritional status. The individual variance in reactivity to stress shown by the mice as well as the breeding hierarchy formed may affect several acute parameters immediately after isolation. We discussed our hypothesis in relation to changes to parameters in a certain direction to such an extent as to surpass the nutritional variance by hierarchy.

Chronic stress loading by social defeat [13, 22] or mild restraining [7] also increased food intake and the preference for palatable food, but it should be noted that all of these findings were obtained using young mice and they agreed well with our own results, where increases in food intake were observed only in young mice and not in aged mice. Therefore, continuous mild stress is likely to increase food intake in young mice.

Under stress, glucocorticoids are known to affect feeding behavior in addition to playing a role in negative feedback to the stress response [8]. In contrast to our expectations, we found no significant differences in the plasma corticosterone levels of the group-housed and isolated mice in both the young and aged groups after 2 weeks of isolation (S1 Fig). According to our previous study with young mice, the plasma corticosterone levels increased temporarily with a peak at 30 min after isolation, before returning to the normal level [1]. Thus, after 2 weeks of isolation, the corticosterone levels appeared to have already returned to the normal level, or they did not increase (S1 Fig). This suggests that the increased food intake under subchronic isolation is unlikely to be due to an excess of glucocorticoids.

It has been demonstrated that excitation of the sympathetic nervous system due to stress loading increases the body temperature, blood pressure and heart rate, thereby leading to excessive energy expenditure [23–25]. The young mice used in the present study were in the growth stage and their weight increased on a daily basis. In this experiment, we detected no difference in the body weight gain between the group-housed and isolated groups, despite the increased food intake by the isolated group. This suggests that the energy balance was maintained by increased energy expenditure in the young isolated mice. By contrast, the body weights of the aged male mice decreased significantly in the isolated group, while the body weights also tended to decrease in the isolated female mice. Brown adipose tissue largely controlled by the sympathetic nervous system has the ability to dissipate energy in the form of heat through the actions of uncoupling protein–1, thereby critically influencing energy expenditure. In the recent study, an association energy expenditure and thermogenic marker in the stress models has been reported [26, 27]. In the current study, while we did not investigate effect of isolation on these factors, we measured the rectal temperature in young male and female mice during isolated housing in the preliminary test. Contrary to expectations, even at 24 h and 2week after isolation should be having the highest stress response, a change in apparent temperature was not observed (Data not shown). From the results, it was considered to be a low possibility of increasing the energy consumed by the increase in deep body temperature in young mice during isolation. We should investigate a further examination to measure skin temperature or thermogenic molecular in young and aged mice.

Interestingly, the aged mice exhibited no significant isolation-induced increase in food intake and they had lower locomotor activity levels in the second week of isolation compared with those of the young mice. These results suggest that food intake regulation, which is an essential energy intake mechanism for body weight maintenance, does not function fully in aged mice in the subchronic stress model, regardless of gender. A future study should elucidate the energy metabolism process during isolation to further validate this hypothesis.

To examine the effects of gender and aging on food intake during isolation, we analyzed the plasma ghrelin levels and gene expression levels in the hypothalamus of mice subjected to 18-h fasting. This experiment was performed after 1 week of isolation in order to identify the causes but not the results of the change in food intake. The plasma acylated ghrelin levels in young isolated male mice were significantly higher than those in the control. In young male mice, isolation caused significant increases in the hypothalamic expression levels of NPY, AGRP, and orexin genes (S2 Fig), which are indices of peripheral ghrelin signaling. These results suggest that the isolation-induced elevation of peripheral acylated ghrelin was transmitted as a signal to the hypothalamus. It has been reported that the acylated ghrelin levels in the plasma increase in water avoidance stress [28], tail pinch stress [29], and social defeat models [5, 12, 13], where the food intake increase in these stress models is mediated by NPY [30]. These studies were conducted using young male mice and they agreed with the results of our study. Thus, in young male mice, it is considered that the isolation-induced peripheral acylated ghrelin increase mediated the enhanced feeding behavior during isolation.

In young female mice, food intake was enhanced by isolation, as found in young male mice. However, there were no increases in the plasma acylated ghrelin levels or the expression levels of appetite-promoting genes in the hypothalamus, but instead the AGRP gene expression level was decreased significantly by isolation. These findings contrast with the results obtained in young male mice, thereby suggesting that factors other than peripheral ghrelin or hypothalamic appetite-promoting genes contribute to the enhanced feeding behavior observed in isolation stress-loaded young female mice. At present, we have no direct evidence to explain the discrepancy between enhanced feeding and negatively regulated peripheral ghrelin signaling in young female mice. A possible explanation is the activation of hypothalamic ghrelin neurons, the functions of which have not been fully elucidated [31]. In the present study, there was no difference in the peripheral ghrelin levels in young female mice after 1 week of isolation and those in the group-housed mice; however, in contrast to the male mice, the preproghrelin mRNA expression level increased significantly in the hypothalamus compared with that in the group-housed mice. The gene expression levels in different areas of the brain were not evaluated in the present study, but ghrelin synthesis in the hypothalamus appears to have been elevated during isolation in the young female mice.

Isolation caused a significant increase in the plasma acylated ghrelin level in aged male mice, but this increase was slight and it had no effect on the A/D ratio (S1 Fig). Furthermore, there were no changes in the expression levels of the NPY, AGRP, and orexin genes in the aged mice during isolation. These findings suggest that dysregulation of ghrelin secretion and ghrelin resistance occurred in the appetite control system of aged mice, which agrees with our previous results [16].

In general, the locomotor activity level increases with motivation and exploratory behaviors, such as food seeking [32] or environmental stimuli [33]. The locomotor activity in rodents increases significantly after the start of the dark phase alongside the increase in feeding behavior, before decreasing gradually toward the light period [34]. In the present study, this typical activity change, which is consistent with the diurnal rhythm, was seen in the group-housed young mice. However, to our surprise, the locomotor activity levels of young female mice increased remarkably when they were kept in isolation compared with that in the young male mice. Activation of the CRF receptor [35] or orexin receptor [36] has potent stimulatory effects on behavioral arousal and locomotor activity, thereby leading to anxiety-like behavior or searching behavior. The expression of these genes resulted in significant decreases or decreasing trends in isolation (S2 Fig), so it is conceivable that the isolation-induced increase in the locomotor activity of young female mice was not due to the activation of the CRF or orexin receptor, but instead it might simply have been due to the anxiety caused by exposure to isolation stress.

Locomotor activity is closely related to the reward system, which is also the case with feeding behavior, and dopaminergic neurons are well known to play a central role in these processes. Recently, cerebral ghrelin has been shown to mediate the dopaminergic neuron reward system [37–40]. In the hypothalamus, ghrelin may act on the dopaminergic neurons in the ventral tegmental area (VTA) [41], as well as facilitating the release and turnover of dopamine [40]. The direct administration of ghrelin to VTA, the neurons of which project from the lateral hypothalamus and arcuate nucleus, is known to increase locomotor activity [37, 39]. This suggests that there is a clear association between locomotor activity and stimulation of the reward system with intracerebral ghrelin. In the present study, the preproghrelin mRNA expression level increased significantly in the hypothalamus of young female mice after isolation compared with that in the group-housed mice. Therefore, it is possible that the increased expression of the hypothalamic preproghrelin gene contributed to the increased locomotor activity observed in subchronically isolated young female mice. By contrast, in young male mice, the preproghrelin mRNA expression level in the hypothalamus decreased significantly, but without changes in locomotor activity. Compared with the young mice, there were no major changes in the preproghrelin mRNA expression levels in the hypothalamus or in the diurnal locomotor activity levels in both the group-housed and isolated groups of aged mice. Unlike the isolated aged male mice, the locomotor activity levels of isolated aged female mice only increased during the light period, but the basal level was lower than that in the young mice. Detailed reason on the hyperactivity of the isolated aged female mice is unknown. It is considered that a decrease in locomotor activity due to aging may have a larger physiological significance than significant hyperactivity of female mice during the light phase, and that female mice tend to easily show increased locomotor activity under stress conditions such the isolation. Various factors are likely to be involved in the decreased locomotor activity levels of aged mice. These factors may include leptin, a counterpart of ghrelin, which is known to negatively control dopamine release and dopamine-activated locomotor activity [42]. It has been shown that aged mice are in a condition of hyperleptinemia due to increased abdominal fat [16]. In the present study, the leptin level was elevated in aged mice after 2 weeks of isolation with free access to food, which supports previous results (data not shown). These findings suggest that the decrease in the locomotor activity levels of aged mice may be mediated by excess peripheral leptin.

Changes in food intake and locomotor activity levels, which are closely associated with the reward system, may be related to changes in stress avoidance psychological behaviors during isolation rearing, such as coping and adaptation to stress; thus, the increased peripheral ghrelin levels in young male mice might mediate stress adaptation, thereby determining their feeding behavior in the present study. Unlike the male mice, increases in peripheral ghrelin were not seen in young female mice, but their food intake increased and their body weight was maintained. The reason for this is not clear, but increased ghrelin secretion from the hypothalamus might have compensated for the decreased peripheral ghrelin level. A reciprocal relationship between peripheral ghrelin and that in the hypothalamus has been reported previously [43]. In addition, increased ghrelin in the hypothalamus may have contributed to the increased locomotor levels in the young female mice. In the aged mice, there were dysfunction in the peripheral ghrelin and in the hypothalamus ghrelin, and it was thought that it caused a decrease in stress adaptation.

**Fig 6:**
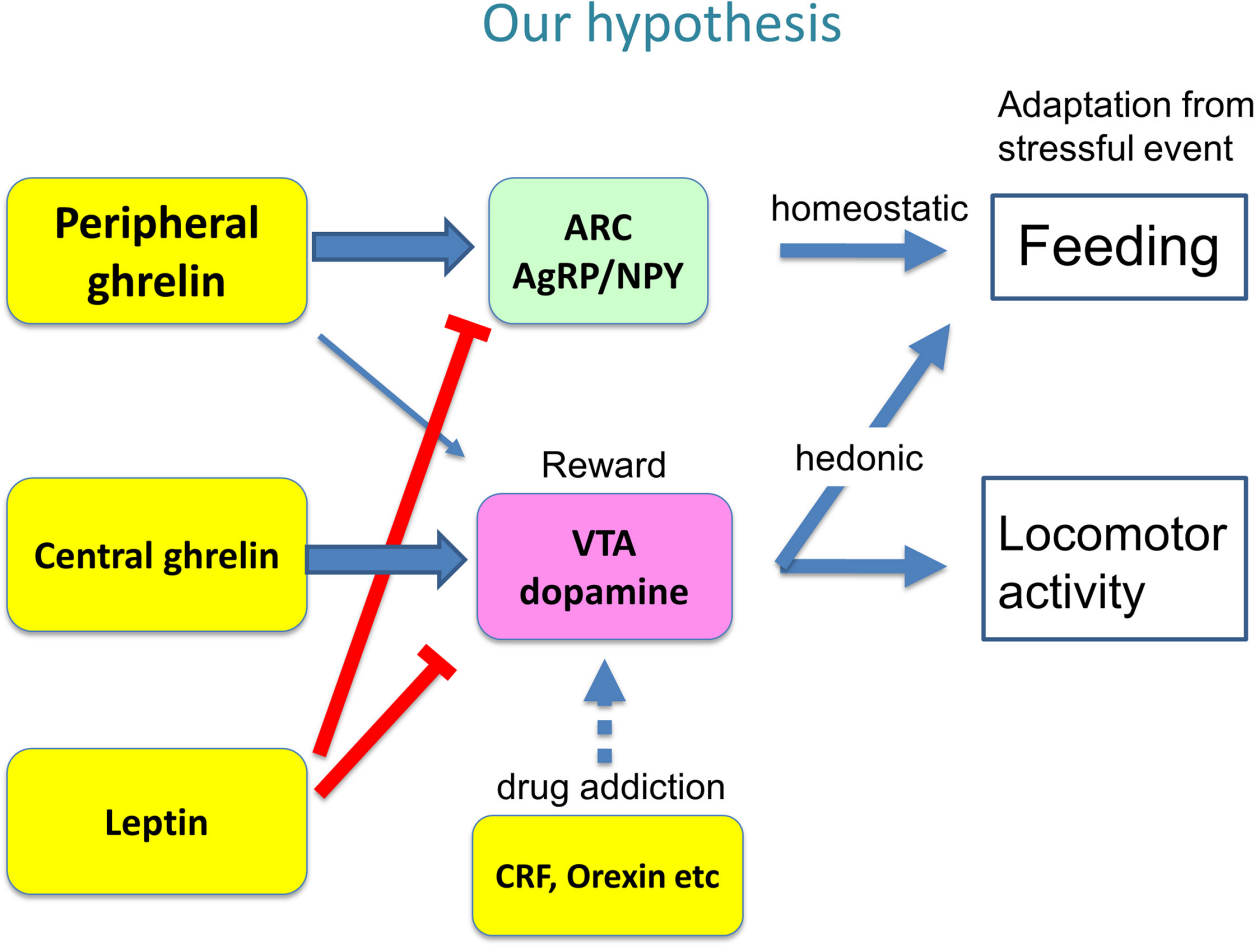
Possible mechanisms for age- and gender-specific changes in feeding and locomotion after subchronic isolation.

Based on the results obtained in the present study, we hypothesize possible mechanisms for age- and gender-specific changes in feeding and locomotion after subchronic isolation, as shown in Fig 6. Further detailed studies will be required needed to test our hypothesis, in the future.

This study is the first report that focuses on gender and age of subchronic isolation stress. Sex hormones such as testosterone and estrogen also may influence the stress reaction and feeding behavior [18, 44]. Our experiments have been performed during the spontaneous estrus cycle, although we did not intentionally align the period of the experiment with the cycle. In the previous reports, we performed evaluations of testosterone and estrogen levels in the blood of both young and aged mice after isolation [3]. Since a significant change was not also observed in the sex hormone levels between all groups in this study (Data not shown), we believe that the influence of the estrous cycle on the parameters may be marginal.

## Conclusion

Unlike acute stress responses, subchronic isolation stress increased food intake in young mice but not in aged mice. Gender- and age-induced functional changes related to peripheral and central ghrelin kinetics, as well as adaptation to an isolated environment, are possible mediators of this response.

## Supporting Information

S1 Fig Changes in the plasma A/D ratio after 1 week of isolation and the plasma corticosterone levels after 2 weeks of isolation. After 1 week of isolation, the mice were fasted for 18 h. After 2 weeks of isolation, the mice fed freely. The acylated ghrelin/des-acyl ghrelin (A/D) ratio after 1 week and plasma corticosterone levels after 2 weeks are shown in young male mice (A,E), young female mice (B,F), aged male mice (C,G), and aged female mice (D,H). The data represent the mean ± SEM (n = 4–9). *, **p <0.05, 0.01 respectively vs. group-housed mice.
(TIF)

S2 Fig Changes in orexigenic gene expression after 1 week of isolation. After 1 week of isolation, the mice were fasted for 18 h. The gene expression levels are shown in young male mice (A), aged male mice (B), young female mice (C), and aged female mice (D). The data represent the mean ± SEM (n = 4–8). *, **, ***, *p* <0.05, 0.01, 0.001 respectively vs. group-housed mice.
(TIF)
