# Supplementary figures and images for: Influence of Aging and Gender Differences on Feeding Behavior and Ghrelin-Related Factors during Social Isolation in Mice
Source: PLoS One. 2015 Oct 8;10(10):e0140094. doi: 10.1371/journal.pone.0140094 (PMC4598162; doi:10.1371/journal.pone.0140094)

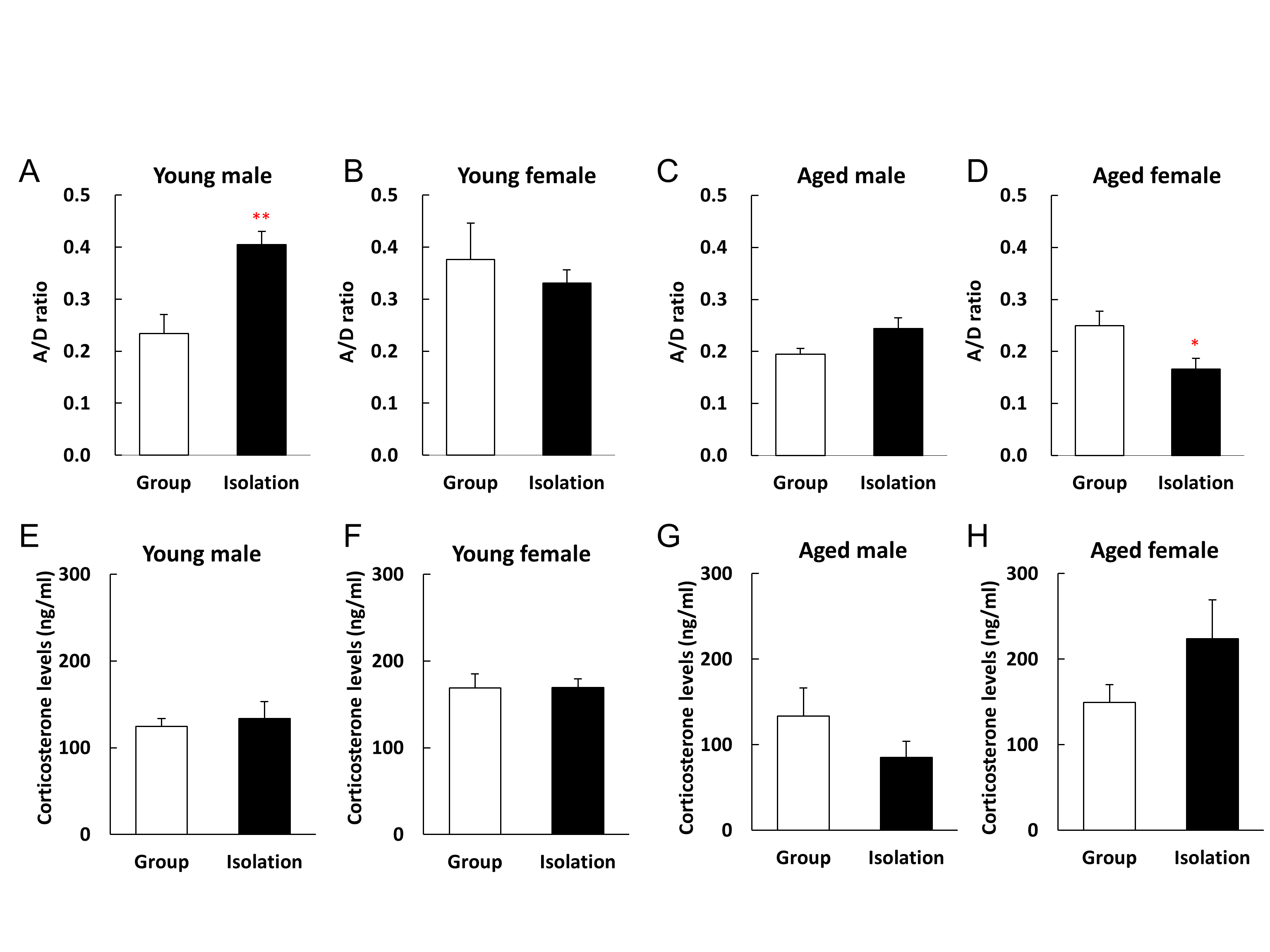

Supplement: S1 Fig — After 1 week of isolation, the mice were fasted for 18 h. After 2 weeks of isolation, the mice fed freely. The acylated ghrelin/des-acyl ghrelin (A/D) ratio after 1 week and plasma corticosterone levels after 2 weeks are shown in young male mice (A,E), young female mice (B,F), aged male mice (C,G), and aged female mice (D,H). The data represent the mean ± SEM (n = 4–9). *, **p <0.05, 0.01 respectively vs. group-housed mice. (TIF) [file pone.0140094.s001.tif]

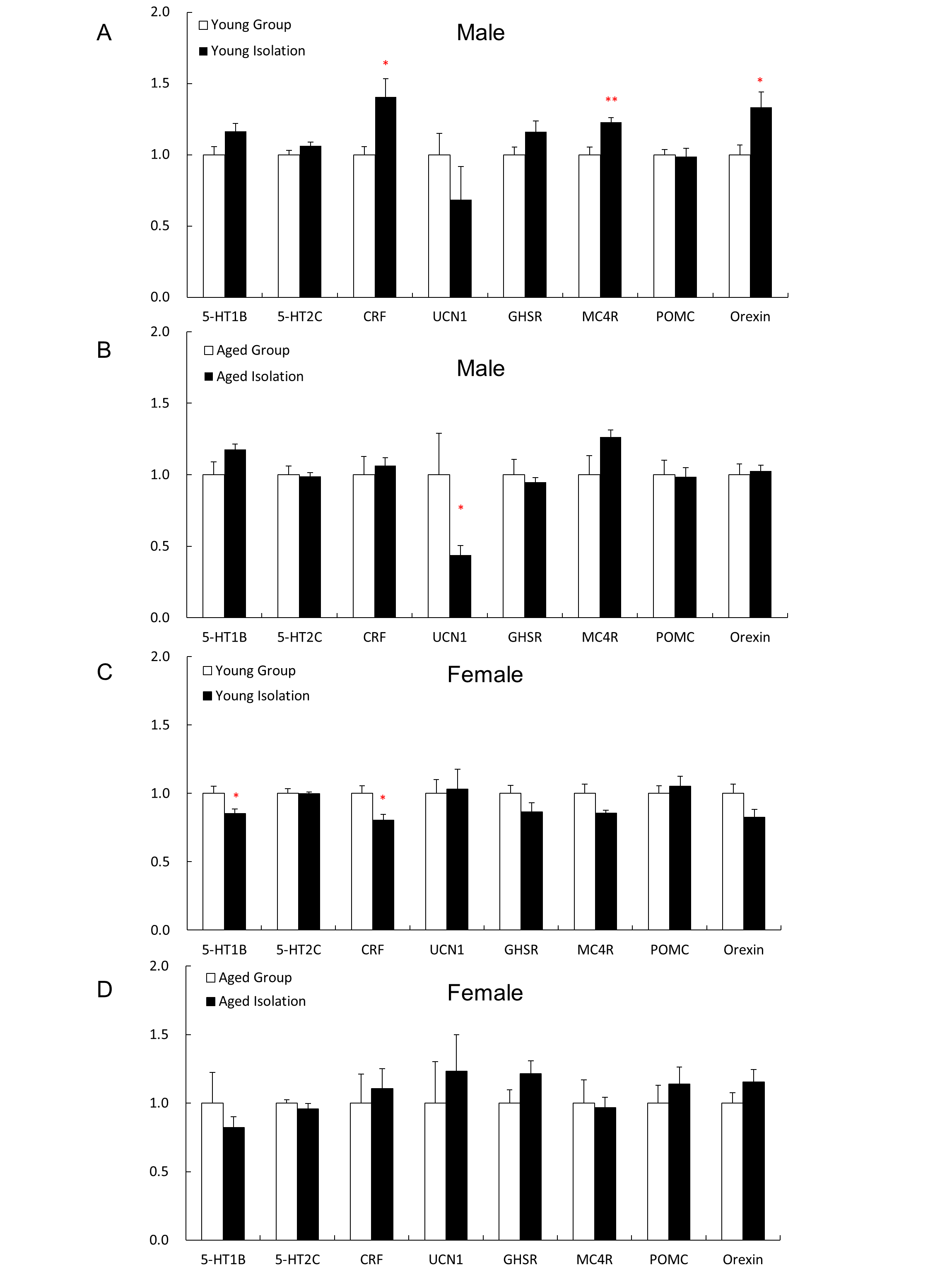

Supplement: S2 Fig — After 1 week of isolation, the mice were fasted for 18 h. The gene expression levels are shown in young male mice (A), aged male mice (B), young female mice (C), and aged female mice (D). The data represent the mean ± SEM (n = 4–8). *, **, ***, p <0.05, 0.01, 0.001 respectively vs. group-housed mice. (TIF) [file pone.0140094.s002.tif]
